# Sensitizing the Therapeutic Efficacy of Taxol with Shikonin in Human Breast Cancer Cells

**DOI:** 10.1371/journal.pone.0094079

**Published:** 2014-04-07

**Authors:** Wenjuan Li, Joan Liu, Kasey Jackson, Runhua Shi, Yunfeng Zhao

**Affiliations:** 1 Department of Pharmacology, Toxicology & Neuroscience, LSU Health Sciences Center in Shreveport, Shreveport, Los Angeles, United States of America; 2 Feist-Weiller Cancer Center, LSU Health Sciences Center in Shreveport, Shreveport, Los Angeles, United States of America; 3 School of Basic Medicine, Hebei University, Baoding, Hebei, China; The University of Tennessee Health Science Center, United States of America

## Abstract

Shikonin, a small-molecule natural product which inhibits the activity of pyruvate kinase M2 (PKM2), has been studied as an anti-cancer drug candidate in human cancer models. Here, our results demonstrate that shikonin is able to sensitize human breast cancer cells to chemotherapy by paclitaxel (taxol). Human breast adenocarcinoma MBA-MD-231 cells, which have higher levels of PKM2 expression and activity compared with MCF-7 cells, were selected to study further. The concentrations of shikonin and taxol were first selected at which they did not significantly induce cytotoxicity when treated alone, whereas the combination induced apoptosis. Surprisingly, PKM2 activity was decreased by shikonin, but not by the combination treatment. To identify the potential targets of this combination, human phospho-kinase antibody array analysis was performed and results indicated that the combination treatment inhibited the activation of ERK, Akt, and p70S6 kinases, which are known to contribute to breast cancer progression. Finally, how the combination affects breast cancer cell growth *in vivo* was tested using a xenograft tumor model. The results indicated that shikonin plus taxol prolonged animal survival and reduced tumor size than the vehicle treatment group. In summary, our results suggest that shikonin has a potential as an adjuvant for breast cancer therapy.

## Introduction

Shikonin is a natural product isolated from the plant *Lithospermum erythrorhizon*, which has long been used as a traditional Chinese medicine [1]. The therapeutic effects of shikonin range from anti-microbial, anti-inflammatory, and anti-tumor effects. Early studies have revealed the various mechanisms of shikonin-induced cancer cell stress. For instance, shikonin induces apoptosis in breast cancer cells, and it also inhibits tumor proteasome activity both *in vitro* and *in vivo*, which prevents the degradation of tumor suppressor proteins [2]. Shikonin can inhibit the activities of DNA topoisomerases, which plays a crucial role in cancer cell DNA regulation, including replication, recombination, and transcription [3]. Other mechanisms of shikonin-induced cancer cell death include increased expression of p53 and inhibition of cancer cell glycolysis via targeting pyruvate kinase M2 (PKM2) [4], [5].

Taxol (paclitaxel) is a widely used chemotherapeutic drug for various types of human cancers. The drug works by stabilizing microtubules to induce cell cycle arrest and prevent cell proliferation [6]. Studies have shown that taxol induces cell cycle arrest in breast cancer cell line MDA-MB-231 at the G2/M phase [7]. Although taxol has been successfully used as an anti-cancer drug, rising drug resistance has created a drawback.

Utilizing natural compounds such as shikonin as an adjuvant for chemotherapy may help overcome the toxicity and drug resistance. For this purpose, this study looks into the combination of shikonin and taxol.

Furthermore, since shikonin is a known PKM2 inhibitor [7], it will be interesting to study if targeting cancer cell metabolism by shikonin can sensitize taxol, which acts on a totally different pathway.

The Warburg Effect, a metabolic characteristic of cancer cell metabolism, describes that cancer cells primarily utilize glycolysis and lactic acid fermentation for energy production, rather than oxidation of glycolytic end products through the Krebs cycle [5]. PKM2 is a vital part of the last rate-limiting step in glycolysis, making it an attractive target as a new chemotherapeutic strategy [4]. In this study, whether targeting PKM2 by shikonin in PKM2 highly expressed breast cancer cells can sensitize these cells to taxol treatment will be examined.

## Materials and Methods

### Cell cultures and chemicals

Human breast cancer MCF-7 and MDA-MB-231 cells were purchased from American Type Culture Collection, Manassas, VA. Cells were grown in Dulbecco's Modified Eagle Medium (DMEM) with 10% fetal bovine serum and in a humidified environment at 37°C under 5% CO_2_. Taxol was purchased from Enzo Life Sciences (BML-T104), and shikonin was purchased from Sigma (S7576). Both chemicals were dissolved in dimethyl sulfoxide (DMSO).

### MTT viability assay

Inhibitory effects of varying dosages of taxol and shikonin were measured using cell viability assay. Cells were seeded in a 96-well plate at 1×10^4^ cells per well overnight. Cells were treated with either taxol, or shikonin, or the combination, and incubated for 24 h at 37°C. MTT (0.5 μg/μl, purchased from Sigma) was added to each well and incubated for 4 hours at 37°C, and the medium was replaced with 100 μl 0.01 N HCl and 10% SDS mixture and incubated for 30 min at room temperature. Absorbance was measured with a microplate reader at 655 nm wavelength.

### Preparation of whole cell lysate

Collected cells were rinsed with cold PBS and suspended in PBS containing a protease inhibitor cocktail. Cells were sonicated on ice for 3 strokes at 15 s per stroke. Samples were incubated on ice for 30 minutes and then centrifuged for 15 minutes at 12,000 rpm at 4°C. Supernatants were collected as whole cell lysate for PKM2 activity assay. For western blot analysis, cell lysate was prepared using RIPA cell lysis buffer (Santa Cruz Biotechnology).

### Western blot analysis

Whole cell lysate was used for the assay. Antibodies against PKM2 (ab38237) and GAPDH were purchased from Abcam and Santa Cruz Biotechnology, respectively. ECL reagents (Thermo Scientific) were used for visualization of bands.

### PKM2 activity assay

The total volume of assay mixture for one sample was 400 μL which consisted of the following: 1 M tris-acetate pH 7.0, 3 M KCL, 1 M MgCl_2_, 100 mM ADP, 10 mM NADH, and LDH. 5 μL whole cell lysate was added to the assay mixture. The change in absorbance at 340 nm was recorded for 1 minute. In addition to cell lysate and phosphoenolpyruvate (PEP), 5′-AMP (1 mmol/L) was also added to the assay mixture to measure PKM2 activity.

### Apoptosis assay

The caspase assay kit was purchased from G-Biosciences. Whole cell lysate was incubated with the assay buffer (containing DTT) and AFC-conjugated substrate. Samples were incubated at room temperature and fluorescent signals were detected after incubation periods of 0, 30, and 60 minutes (360±40 nm for excitation and 528±20 nm for emission).

For annexin V staining, MDA-MB 231 cells were seeded at 1×10^6^ cells per plate in 100 mm tissue culture plates. The cells were incubated at 37°C in a 5% CO_2_ incubator overnight. Cells were treated for 24 h with Taxol (8 nM) and Shikonin (2.5 μM). Apoptosis was determined using Annexin V (A13199, invitrogen, Carlsbad, CA) and PI (195458, MP Biomedicals, Solon, Ohio) following the instructions provided by the manufacturer. In brief, cells were washed twice with cold PBS, then add annexin V and incubate at room temperature for 15 min. Add PI just before detection, apoptosis was determined using the BD Biosciences flow cytometer. Data were gathered using the ModFit LT software.

### Xenograft tumor studies

The animal study was performed under the approved animal protocol by the Institutional Animal Care & Use Committee of LSU Health Sciences Center in Shreveport. 6-8-week-old nude mice (athymic nu/nu, female, Charles River) were randomly separated into 3 groups (7-11 per group). Each group received a subcutaneous injection of 2×10^6^ MDA-MB-231 cells into the fat pad. Tumor size (biggest dimension × smallest dimension^2^ ×0.52) and animal weight were measured daily. When the tumor volume reached approximately 300 mm^3^ (which took approximately one week), mice in Group 1 and 2 received a single i.p. injection of DMSO and taxol (6 mg/kg body weight) [8], respectively; Group 3 received a single i.p. injection of combination of taxol (6 mg/kg body weight) with shikonin (1 mg/kg body weight). Tumor size and animal weight were continually measured daily and mice were euthanized when the tumor volume reached 2,000 mm^3^ by injecting overdosed pentobarbital (150 mg/kg).

### Phospho-kinase array

Relative protein phosphorylation levels were obtained by profiling of 46 specific phosphorylation sites using the Proteome Profiler Human Phospho-Kinase Array Kit (ARY003B, R&D Systems). Experiments were performed according to the instructions provided by the manufacturer. Briefly, cells were rinsed with PBS, resuspended in lysis buffer (1×10^7^ cells per ml), and shaked at 4°C for 30 minutes. Membranes with spotted catcher antibodies were incubated with diluted whole cell lysates at room temperature for one hour. Next, cocktails of biotinylated detection antibodies were added and samples were incubated at room temperature for 2 hours. Phosphorylated proteins were revealed using streptavidin-HRP/chemiluminescence substrate (sc-201696, Santa Cruz biotechnology) and autoradiography films. The resulting spots were scanned and images were quantified using the Image lab software (Bio-Rad) and Microsoft Excel software.

### Statistical analysis

One-way ANOVA followed by Newman-Keuls post-test was used for multi-group comparisons. All of the experiments have been repeated at least three times. p<0.05 was considered significant.

## Results

### Comparison of PKM2 expression/activity levels between MCF-7 and MDA-MB-231 cells

**Figure 1:**
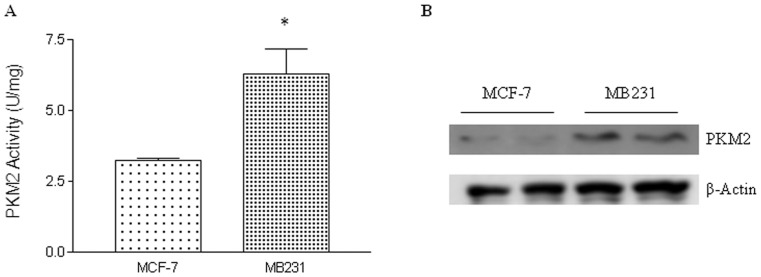
Enzymatic activity and expression levels of PKM2 were detected in both human breast cancer MDA-MB-231 (MB231) cells and MCF-7 cells. Whole cell lysate was subjected to activity and Western blot analysis to determine the enzymatic activity (A) and protein expression (B) of PKM2. β-Actin served as the loading control. * *P*<0.05 compared with MCF-7 cells.

MDA-MB-231 cells are a more aggressive type of breast cancer cell line than MCF-7 cells. The levels of PKM2 activity (A) and expression (B) were detected and the results were shown in Fig. 1. Both the activity and expression levels of PKM2 were approximately 2-fold higher in MDA-MB-231 cells than that in MCF-7 cells. Therefore, MDA-MB-231 cells were selected for the following studies.

### Determination of cytotoxicity of shikonin and taxol using the MTT assay

**Figure 2:**
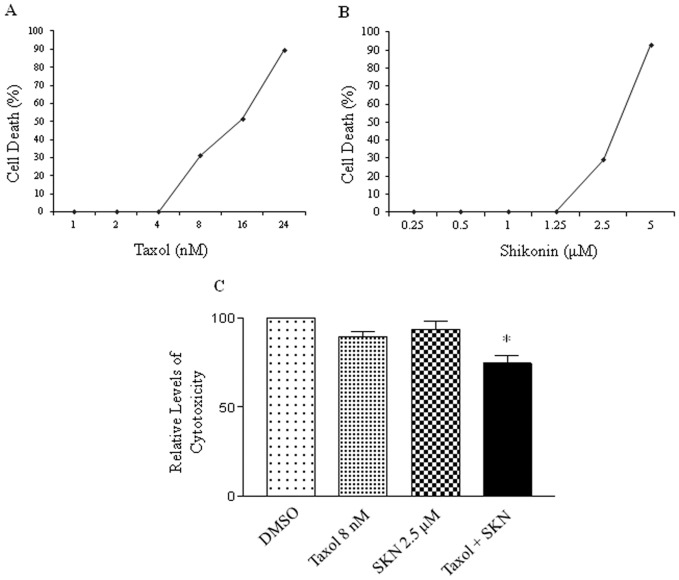
Cytotoxicity assay of the combination treatment. MTT assays were performed to select the concentrations of taxol (A) and shikonin (B). C, cytotoxicity measurement of the combination of taxol and shikonin. The experiments have been repeated more than three times. * p<0.05 when compared to the control (DMSO).

In order to test whether shikonin can sensitize MDA-MB-231 cells to taxol treatment, MTT assays and combination index analysis (Table S1) were performed to select the optimal concentrations of these two agents which also show synergism when combined. As shown in Fig. 2A and 2B, the calculated IC50 values were 14.5 nM for taxol and 3.5 μM for shikonin. Based on the IC50 values, the dosages of 8 nM for taxol and 2.5 μM for shikonin were selected for the combination treatment to determine if shikonin could enhance the growth inhibition of taxol in breast cancer cells. The result in Fig. 2C indicates that the combination of taxol and shikonin generated a synergistic growth inhibition of MDA-MB-231 cells.

### Effects of the combination treatment on PKM2 activity

**Figure 3:**
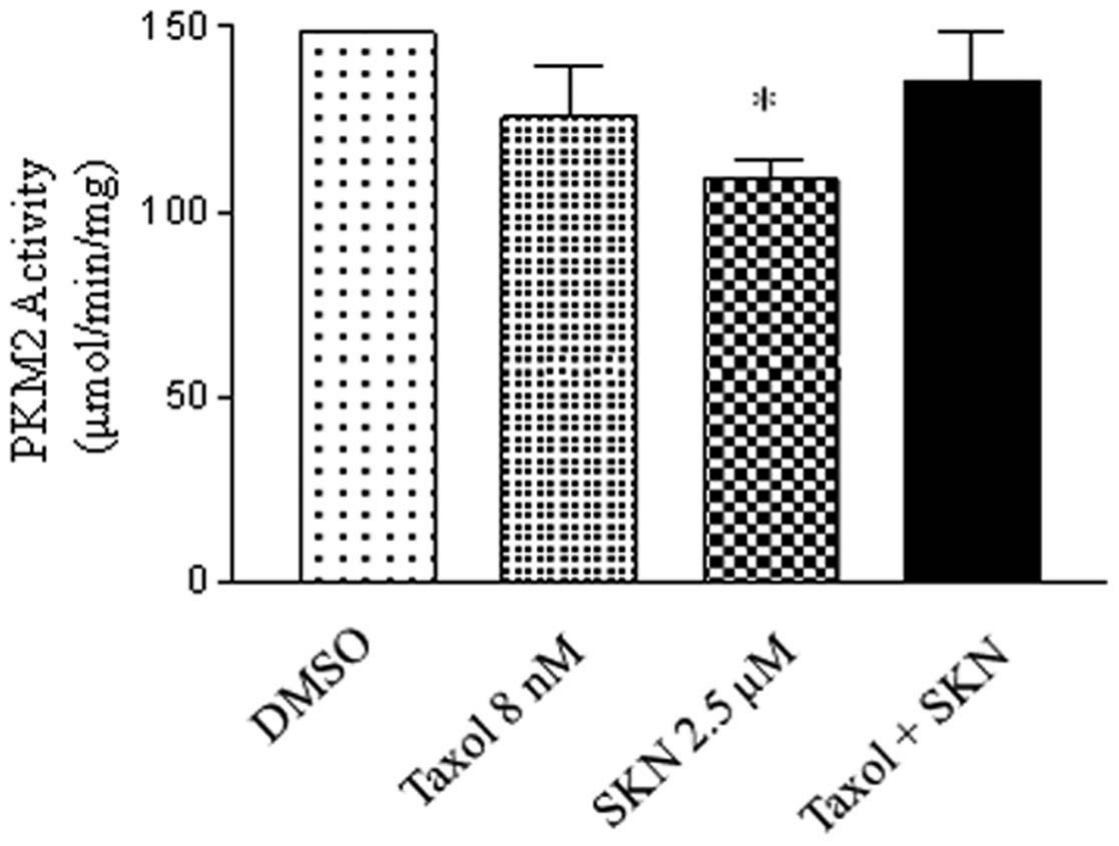
Enzymatic activity of PKM2 was measured from cells treated with DMSO as the vehicle control, taxol (8 nM), shikonin (2.5 μM), and the combination. *, p<0.05 compared to the control group. The experiments have been repeated more than three times.

To study the effects of the combination (taxol plus shikonin) treatment on glycolysis, the activity levels of PKM2 were measured and compared between individual and combination treatments. As summarized in Fig. 3, the inhibition of PKM2 activity by shikonin alone was significant; neither taxol nor the combination treatment caused a significant decrease in PKM2 activity. These results indicate that the combination treatment does not act on PKM2.

### Shikonin and taxol treatment induces apoptosis

**Figure 4:**
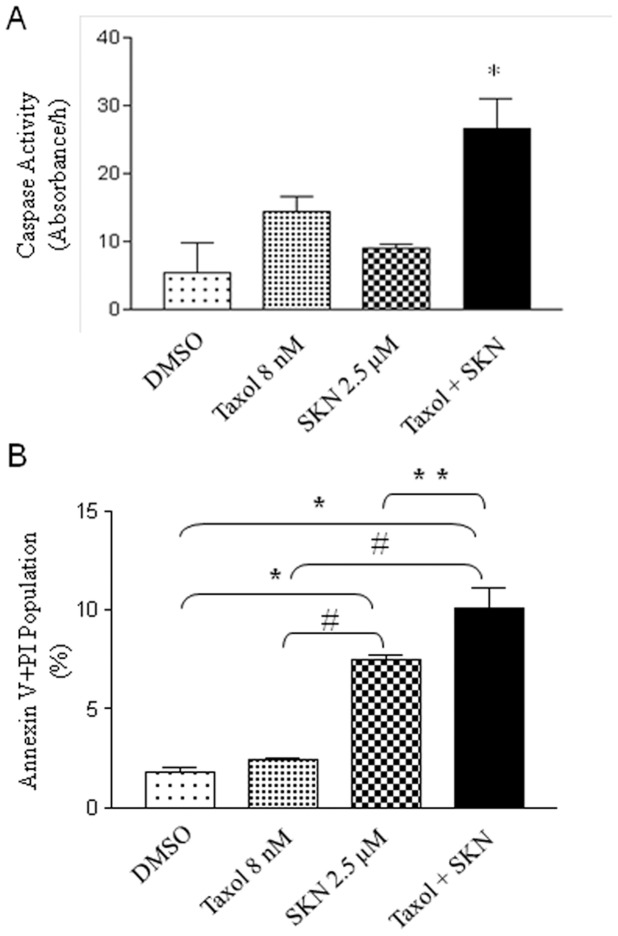
Apoptosis analysis of the combination treatment. Both caspase activity (A) and annexin V conjugates for apoptosis (B) were measured in MDA-MB-231 cells treated with DMSO as the vehicle control, taxol (8 nM), shikonin (2.5 μM), and the combination. Activity was measured as Δabsorbance after 1 hr incubation with the DTT assay buffer and the AFC-conjugated substrate (n = 3 in each group). * P<0.05 when compared to the control (DMSO), # *P*<0.05 when compared to the Taxol; ** *P*<0.05 when compared between SKN and Taxol+SKN.

To detect if the combination treatment induces a greater level of apoptosis, activities of caspase 3, 7, and 10 were first measured. MDA-MB-231 cells treated with selected dosages from the cell viability assay were used for measuring the caspase activity. As shown in Fig. 4A, neither taxol nor shikonin induced significant increases in caspase activity; the combination treatment induced a 9-fold increase in caspase activity compared with the vehicle treatment, which is also statistically significant. Apoptosis was further assessed by annexin V and PI staining (Fig. 4B). Cells were treated similarly as above mentioned, and subjected to flow cytometry analysis. The result indicated that the combination treatment significantly induced apoptosis.

### The taxol and shikonin combination inhibits the activation of Akt and ERKs

**Figure 5:**
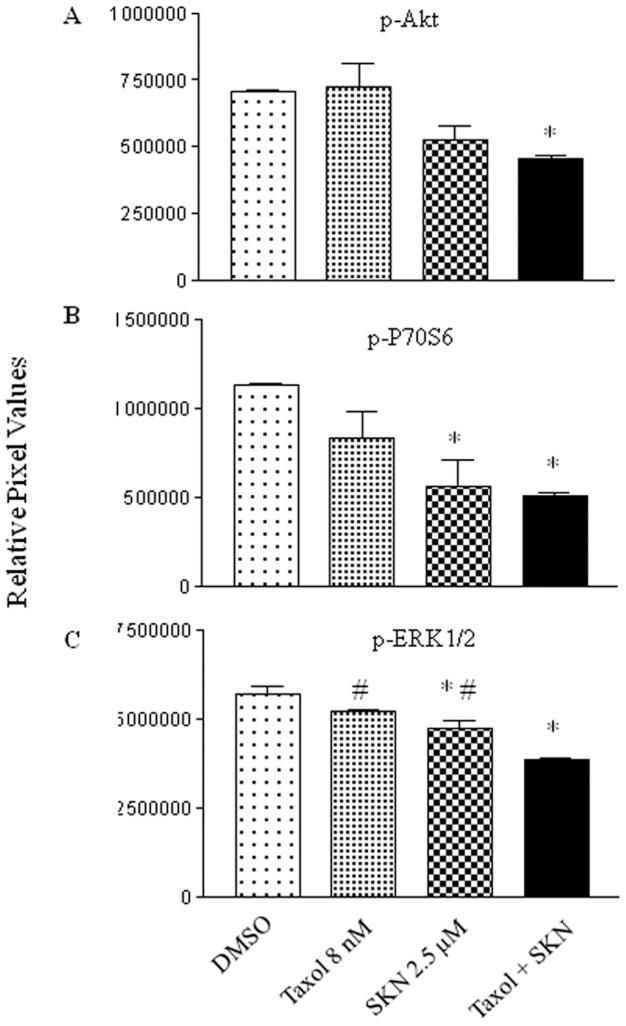
The effect of the combination treatment on key pro-survival kinases. The combination of taxol and shikonin inhibites the activation of Akt (A), p70S6 (B), and ERKs (C). Human phosphor-kinase array analysis was performed following the manufacturer's instruction. Whole cell lysate from MB231 cells treated with DMSO, taxol (8 nM), shikonin (2.5 μM), or the combination. The normalized intensity for each antibody was plotted. * p<0.05 when compared to the control (DMSO); # p<0.05 when compared to the combination.

To address the potential molecular mechanisms underlying the synergism of the taxol-shikonin combination, a human phosphor-kinase array assay was utilized. This technique is a powerful mechanism for gaining a global view of changes in signal transduction events within cells. It has been known that Akt plays an important role in confirming the chemosensitivity of many human cancer cells [9]–[11]. In this study, as shown in Fig. 5A, the combination treatment inhibited the activation of Akt by reducing its phosphorylated form (T308). In addition, the combination decreased the levels of phosphorylated p70S6 (Fig. 5B), which is a downstream target of Akt. Furthermore, the combination treatment inhibited the phosphorylation of ERKs (Fig. 5C), which are involved in the processes of cell proliferation, differentiation, inflammation, survival and transformation [12]–[14].

### The effect of the combination treatment on survival probability *in vivo*

**Figure 6:**
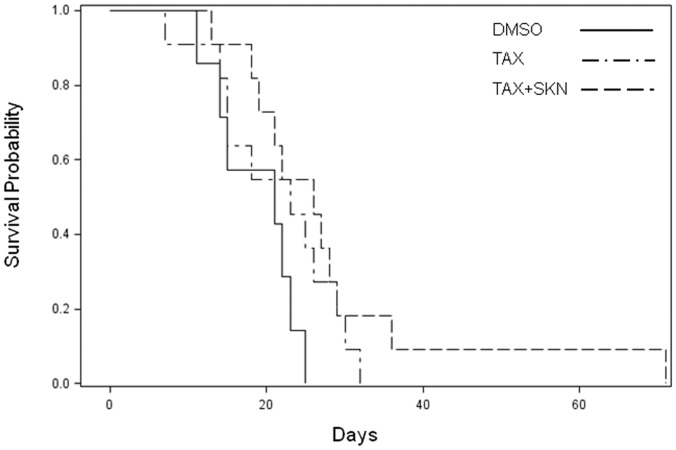
Survival probability of MD-MB-231 tumor xenografted mice. MD-MB-231 cells were transplanted into the animals. When tumor size reached 300 mm^3^, mice were randomized to receive a single i.p. injection of DMSO (n = 7), taxol (6 mg/kg body weight, n = 11), or the combination of taxol and shikonin (1 mg/kg body weight, n = 11). p = 0.0918 when the DMSO and the combination group were compared.

Finally, the therapeutic efficacy of the taxol-shikonin combination was studied in MD-MB-231 tumor xenografts. Mice were treated with vehicle control, taxol, or taxol combined with shikonin. As shown in Fig. 6, monotherapy with taxol was not able to increase survival probability. The combination of taxol and shikonin marginally (p = 0.0918) prolonged survival probability compared with vehicle control.

## Discussion

Targeting cancer cell metabolism has been suggested as a new and promising chemotherapy approach. Since cancer cells rely heavily on glycolysis and PKM2 is the last rate-limiting enzyme in glycolysis, shikonin, a naturally occurred PKM2 inhibitor, might be able to suppress cancer cell growth by inhibiting the main source of ATP production [4]. In addition, shikonin's effect on cellular metabolism may also render its activity to sensitize resistant cells to chemotherapy. Taxol, mediated by microtubule stabilization, is a widely used anti-cancer drug to treat various forms of cancer. Rising drug resistance to taxol poses a problem for future cancer treatment, and the mechanisms of resistance are not clearly understood [15]. In this study, shikonin is tested if it can sensitize MBA-MD-231 cells, a more aggressive type of human breast cancer cells with higher PKM2 activity, to taxol treatment. The dose of each agent is not toxic to the cells under the experimental conditions.

Shikonin itself indeed reduces PKM2 activity by approximately 27%, but it does not show this inhibitory effect when toxal is present. However, the combination does show synergism as demonstrated by the results of caspase activity assay and annexin V staining. There have been controversies on whether PKM2 should be activated or inhibited for cancer therapy [16], [17]. Nevertheless, in this study, the synergy of the taxol-shikonin combination may not rely on inhibiting PKM2.

The kinase array analysis reveals a few potential targets for the combination of taxol and shikonin. Akt binds to phospholipids which is generated via the PI3K pathway and translocates from the cytoplasm to the inner surface of the membrane, and is activated by phosphorylation [18]. Active Akt regulates a number of downstream targets which have effects on survival and cell proliferation, therefore promoting tumor progression [19]. The Akt/p70S6K signal pathway is highly involved in tumorigenesis and metastasis [20], [21]. In this study, the combination of taxol and shikonin inhibits not only Akt but also its downstream p70S6K kinase, whereas this inhibition is not evident by taxol treatment. ERK is another kinase which can induce uncontrolled growth [22], and compounds which inhibit ERK pathway have been testing for cancer treatment. In this study, both taxol and shikonin inhibits ERK activation, and the combination shows a greater effect on ERK inactivation.

Overall, the results from this study demonstrate that shikonin could sensitize aggressive breast cancer cells to taxol treatment. Akt/p70S6K and ERKs might be the target of this combination, and future experiments will be performed to study how the combination of shikonin and taxol regulate these kinases.

## Supporting Information

Table S1 CI values generated by isobologram analyses. The combination index (CI) isobologram method was used to analyze synergism for the combined drug effects. Synergy analyses were performing using CompuSyn Version 1.0 software. CI values of 1, <1, and >1 mean additive, synergistic, or antagonistic effects, respectively.
(DOC)
