# Supplementary material for: Sensitizing the Therapeutic Efficacy of Taxol with Shikonin in Human Breast Cancer Cells
Source: PLoS One. 2014 Apr 7;9(4):e94079. doi: 10.1371/journal.pone.0094079 (PMC3977981; doi:10.1371/journal.pone.0094079)
Supplement: Table S1 — CI values generated by isobologram analyses. The combination index (CI) isobologram method was used to analyze synergism for the combined drug effects. Synergy analyses were performing using CompuSyn Version 1.0 software. CI values of 1, <1, and >1 mean additive, synergistic, or antagonistic effects, respectively. (DOC) [file pone.0094079.s001.doc]

Table S1. CI values generated by isobologram analyses

| Taxol (nM) | Shikonin (uM) | CI |
| --- | --- | --- |
| 4.0 | 1.25 | 0.9525±0.07955 |
| 4.0 | 2.5 | 0.9409 ±0.04982 |
| 8.0 | 1.25 | 1.009 ±0.03890 |
| 8.0 | 2.5 | 0.8290 ±0.01999 |
